# Complete Genome Sequence of Ceftriaxone-Resistant Neisseria gonorrhoeae SS3160, Isolated in Tokyo, Japan

**DOI:** 10.1128/MRA.00886-20

**Published:** 2020-09-24

**Authors:** Hirofumi Miyake, Morika Mitobe, Hiroaki Kubota, Hiroki Takano, Keiko Yokoyama, Kenji Sadamasu

**Affiliations:** a Department of Microbiology, Tokyo Metropolitan Institute of Public Health, Tokyo, Japan; University of Maryland School of Medicine

## Abstract

We report the complete genome sequence of ceftriaxone-resistant Neisseria gonorrhoeae SS3160, harboring the mosaic *penA*-60.001 allele. This Japanese isolate has a unique sequence type (ST), ST13429, which was determined by multilocus sequence typing from the chromosome sequence (2,214,955 bp). It carries two plasmids, pConjugative (39,057 bp) and pCryptic (4,207 bp).

## ANNOUNCEMENT

The emergence and dissemination of ceftriaxone-resistant Neisseria gonorrhoeae strains are serious clinical problems. The acquisition of ceftriaxone resistance by N. gonorrhoeae strains is typically attributed to a mosaic mutation in the penicillin-binding protein encoded by *penA* (1). Ceftriaxone-resistant N. gonorrhoeae FC428, which harbors a unique *penA* allele (*penA*-60.001), was discovered in Japan in 2015. Since then, strains harboring the *penA*-60.001 allele have been reported (2–11). Many of those strains, including FC428, belong to sequence type 1903 (ST1903), based on multilocus sequencing typing (MLST); however, non-ST1903 strains have recently emerged (e.g., 18DG342 from Singapore [8], G97687 from the United Kingdom [10], and F91 from France [11]).

A urine specimen from a male urethritis patient at a venereological clinic in Tokyo, Japan, was cultured on Thayer-Martin selective agar plates (Becton, Dickinson and Co., Franklin Lakes, NJ, USA). This study was approved by the ethics committee of the Tokyo Metropolitan Institute of Public Health (approval number 30kenkenken782). After the identification of N. gonorrhoeae by the rapid identification test HN-20 (Nissui, Tokyo, Japan) and transcription-mediated amplification using the Panther system (Hologic, Marlborough, MA, USA), the MICs of 10 antimicrobial agents against N. gonorrhoeae SS3160 were determined with an Etest (bioMérieux, La Balme-Les-Grottes, France). The results in double-dilution format are as follows: ceftriaxone, 0.5 μg/ml; spectinomycin, 16 μg/ml; azithromycin, 0.25 μg/ml; penicillin, 1 μg/ml; tetracycline, 2 μg/ml; ciprofloxacin, >32 μg/ml; cefuroxime, 4 μg/ml; cefotaxime, 2 μg/ml; cefpodoxime, 8 μg/ml; and ceftazidime, 4 μg/ml.

Total DNA was extracted, using a QIAamp DNA minikit (Qiagen, Hilden, Germany), from SS3160 cultivated on GC II agar plates (Becton, Dickinson) overnight at 37°C. The extracted DNA was used for whole-genome sequencing library preparation using a Nextera XT library preparation kit (Illumina, San Diego, CA, USA) and a ligation sequencing kit (Oxford Nanopore Technologies, Oxford, UK) for sequencing with a MiSeq reagent kit v3 (600 cycles; Illumina) and a MinION R9.4 flow cell (Oxford Nanopore Technologies), respectively. DNA shearing and size selection were not performed prior to the library preparation for MinION sequencing, and the Albacore base caller (Oxford Nanopore Technologies) was used to obtain the MinION raw reads. The number of raw reads and the average read length were 1,584,726 reads and 202 bp, respectively, for MiSeq sequencing, whereas those for MinION sequencing were 169,731 reads and 7,841 bp, respectively. The MiSeq raw reads trimmed with Trim Galore v0.4.3 (https://www.bioinformatics.babraham.ac.uk/projects/trim_galore) with default settings and the MinION raw reads trimmed with NanoFilt v2.6.0 (12) at a quality threshold of 10 were hybrid assembled in Unicycler v0.4.8 using the Galaxy Web platform (https://usegalaxy.org.au) with default settings (13). Three circular contigs, corresponding to the chromosome (2,214,955 bp) and two plasmids, identified as pConjugative (39,057 bp) and pCryptic (4,207 bp), were obtained by Unicycler without additional analysis, including overlap trimming. These sequences were annotated with the DFAST v1.2.4 pipeline (https://dfast.nig.ac.jp) (14) with default settings except for structural annotation using Prodigal v2.6.3 (15) and rotating/flipping of the sequences to start at the *dnaA* gene. The G+C contents of the chromosome, pConjugative, and pCryptic sequences were 52.4%, 48.7%, and 51.6%, respectively.

From the chromosome sequence, MLST determined SS3160 to be a new ST (assigned as ST13429). SS3160 was classified as ST233 by N. gonorrhoeae sequence typing for antimicrobial resistance (NG-STAR) (16), which includes the *penA*-60.001 allele. The N. gonorrhoeae ST determined by N. gonorrhoeae multiantigen sequence typing (NG-MAST) for SS3160 was ST16186 (17).

### Data availability.

The complete genome sequences of SS3160, including the chromosome (AP019853) and the plasmids pConjugative (AP019854) and pCryptic (AP019855), were deposited in the DNA Data Bank of Japan (DDBJ). Raw sequence reads were deposited in the DDBJ Sequence Read Archive (DRA) under accession numbers DRR185756 and DRR185757.
